# Evaluation of public awareness, knowledge and attitudes towards basic life support: a cross-sectional study

**DOI:** 10.1186/s12873-018-0190-5

**Published:** 2018-10-29

**Authors:** Samiha Jarrah, Mahfuz Judeh, Mohannad Eid AbuRuz

**Affiliations:** 1 Faculty of Nursing, Applied Science Private University, Amman, Jordan; 2 Applied Science Private University, Amman, Jordan; 3 Amman, Jordan

**Keywords:** Attitudes, Awareness, Jordan, Knowledge, Life support, Public

## Abstract

**Background:**

Out-of-hospital cardiac arrest is a major cause of mortality worldwide. When basic life support techniques are implemented quickly, the chance of survival is doubled. Therefore, this study evaluated public awareness, knowledge and attitudes towards basic life support in Jordan.

**Methods:**

A descriptive, cross-sectional design with a convenience sample of 300 Jordanian adults aged over 18 years, recruited from three metropolitan areas in the northern, middle and southern regions.

**Results:**

A total of 87 participants (29%) stated that they have received training about cardiopulmonary resuscitation (CPR). Among them, 20 participants (23%) received their training through the media. The highest response rate for cardiac arrest signs was chest pain (*n* = 129, 43%). Participants who received training had greater knowledge of the three signs of consciousness evaluation. The numbers of participants who received training and performed chest compression, mouth-to-mouth ventilation, and both compression and ventilation were higher than those who did not receive training. Overall, 256 participants (88.3%) reported that they would perform CPR on someone from their family without hesitation. The most important concern about performing CPR was making a mistake.

**Conclusions:**

Improving knowledge about cardiopulmonary resuscitation is an important topic, which can be achieved by training the general population. Media can play an important role in this issue.

**Electronic supplementary material:**

The online version of this article (10.1186/s12873-018-0190-5) contains supplementary material, which is available to authorized users.

## Background

Public awareness is of great importance, especially for life-saving information and skills. Globally, many countries emphasize related topics in educational institutions and workplaces. Death from sudden cardiac arrest remains very common worldwide [1, 2]. Approximately 50,000 people per year can survive until professional help arrives when basic life support (BLS) measures are performed properly. Out-of-hospital cardiac arrest (OHCA) causes 350,000 deaths each year in Europe [3]. In the US, the mortality rate due to OHCA is more than 90%, causing 276,000 deaths annually [4]. When the first person to witness OHCA uses an automated external defibrillator (AED), the chance of survival doubles. However, this instrument is used in only 4% of cases [5, 6].

The performance of BLS is usually done in chains that can be improved by educational programs. The success rate of BLS can be improved when it is performed by lay people before professional help arrives. This is usually motivated and enhanced by increasing public knowledge, attitudes and understanding of the application of the BLS techniques. Different studies have been conducted worldwide to ascertain the general level of knowledge, attitudes, and awareness about BLS in community settings. The rate was approximately 70% in Slovenia, which was considered a high percentage, reflecting a particular policy whereby training is an obligatory part of learning to drive in the country [7]. However, the percentage of this country’s population with up-to-date knowledge was very low at 2%. On the other hand, the rate was 78% among teachers in Belgium, demonstrating the impact of teaching [8].

BLS training rates vary worldwide, including the following rates: Washington, DC 78% [9], Poland 75% [10], West Australia 64.1% [11], Japan 58% [12], Turkey 40.3% [1], Ireland 28% [13], New Zealand 27% [14], and Hong Kong 21% [15]. The rates also differ according to the specific area where the study was done. For example, in an urban region of Arizona, the rate was 63.2%, but this dropped to 55.4% in a rural region [16].

Studies evaluating knowledge, attitude and awareness about BLS in Middle Eastern countries, including Jordan, are limited. In a study evaluating knowledge and attitudes towards cardiopulmonary resuscitation (CPR) among university students in Riyadh, Saudi Arabia, it was found that 31% did not have prior CPR knowledge [17]. Of those with previous knowledge, 85% felt that it was inadequate. The most common sources of information were television and movies. Approximately 13% of individuals had encountered a situation that required the use of CPR, while only 14% of them performed it. This was mostly due to lack of knowledge (48.2%). Overall, 88% of students would have liked to learn how to perform CPR. The authors concluded that the general attitude towards CPR was positive, but the knowledge on the topic was insufficient [17]. Thus, more focus should be placed on the improvement of CPR skills. In addition, more studies are needed to assess knowledge and attitudes towards CPR in the community.

## Methods

The purpose of the current study was to evaluate public awareness, knowledge and attitudes towards BLS in Jordan.

**Research Questions: (**1) What is the level of awareness among Jordanian lay people regarding BLS? (2) What is the level of knowledge Jordanian lay people have about BLS? (3) Do Jordanian lay people have positive attitudes towards BLS? (4) Does previous training about BLS have an effect on the level of awareness, knowledge, and attitudes towards BLS?

**Design, Sample, and Setting**: A cross-sectional design with a convenience sampling method was used in this study. The participants who met the following inclusion criteria were included: (a) Jordanian nationality, (b) aged 18 years and above, (c) able to read and write Arabic, (d) signed informed consent, and (e) not graduated from or studying at any medical field colleges (medicine, pharmacy, nursing, dentistry, and laboratory). Those individuals were excluded because their university programs have mandatory courses about BLS training.

Data were collected from three major cities in the northern, middle, and southern areas of Jordan to be representative of the country as a whole. Three research assistants collected the data, one for each area. They approached the participants every day from 8 am to 8 pm in shopping malls and public areas, which are similar in the three cities. The research assistants explained the study and the purposes to the participants, and if they agreed to participate, they signed the informed consent form and answered the questionnaire.

To make sure that the sample size was sufficient, a power analysis was performed using an online G*power sample size calculator. Research questions 1–3 were answered by descriptive statistics. Research question number 4 was answered by chi-squared test. Using this information and the assumptions of a power of .8, type 1 error of 0.05, and a medium effect size, the sample size needed was 107 participants. Therefore, we recruited 300 participants to allow for attrition and dropout.

### Measurement

The researchers designed a questionnaire specifically for this study. This questionnaire was based on the latest American Heart Association (AHA) guidelines for BLS and was written in Arabic [18]. The questionnaire included two parts. Part one collected information about participants’ sociodemographic characteristics (i.e., age, gender, marital status, education, and employment). Part two assessed their knowledge, attitudes and awareness about BLS. This latter part consisted of 22 questions about signs and symptoms of cardiac arrest, previous CPR experience, compression location and rate, if the participants were concerned about making mistakes when performing CPR, and people to whom they would provide CPR (Additional file 1).

### Procedure of data collection and ethical consideration

The study was approved by the Institutional Review Board committee at the Applied Science Private University (Faculty 018). The principal investigator met with all research assistants and explained the purpose of the study and the questionnaire. Research assistants explained the study to the participants. They also ensured the participants that they had the right not to participate and to withdraw at any time. After this explanation, if the participants agreed to participate, they were asked to sign an informed consent form and to complete the questionnaire. The study was completely anonymous and no identifiers were required. Data were coded and entered into a password-protected computer, accessible only to the principal investigator and co-investigators.

### Data analysis

Data analysis was done using SPSS version 21. Description of the sample was performed by descriptive statistics, including n (%) or mean ± SD. A chi-squared test was used to determine if there were differences between those who received training and those who did not regarding the identification of cardiac arrest, action performed when witnessing cardiac arrest, and practical application of CPR. Any *p* value < .05 was considered statistically significant. All statistically non-significant results were reported as not significant (NS).

## Results

### Demographic characteristics

A total of 400 participants were invited to participate, of whom 300 agreed and filled out the questionnaire completely, comprising the sample of the study. The participants were distributed between males and females, with a mean age of 33.55 years. More than half of the sample was married and had a high school and university degree. A detailed description of the participants’ characteristics is presented in Table 1.

**Table 1:** Demographic characteristics of the participants (*N* = 300)

Characteristic	Mean ± SD/ n (%)
Age	33.6 ± 11.0
Gender	
Male	139 (46.3)
Female	161 (53.7)
Marital status	
Married	181 (60.4)
Single	109 (36.3)
Divorced	10 (3.3)
Educational Level	
Elementary school	7 (2.3)
Preparatory school	45 (15)
Secondary school	92 (30.7)
Diploma	44 (14.7)
University	90 (30)
Post graduate	22 (7.3)
Job	
Governmental sector	79 (26.3)
Private sector	86 (28.7)
Self-employed	21 (7.0)
Retired	19 (6.3)
House wife	35 (11.7)
Student	35 (11.7)
Farmer	4 (1.3)
Not employed	21 (7.0)

### CPR training

Among the whole sample, only 87 participants (29%) stated that they had received CPR training. Most of those received their training at school (*n* = 33, 37.9%), followed by university (*n* = 26, 29.9%), and the media (television and internet) (*n* = 20, 23%).

### Signs of cardiac arrest

The response rates for the signs of cardiac arrest are presented in Table 2. The highest rates were for (1) chest pain (*n* = 129, 43%), (2) respiratory standstill (*n* = 119, 39.7%), (c) loss of consciousness (*n* = 114, 38%), and (d) difficulty breathing (*n* = 107, 35.7%).

**Table 2:** Response rates for the signs of cardiac arrest

Sign	^a^n (%)
Chest pain	129 (43.0)
Respiratory standstill	119 (39.7)
Loss of consciousness	114 (38.0)
Difficulty in breathing	107 (35.7)
Cyanosis	76 (25.3)
Discontinuation of circulation	62 (20.7)
Nausea	47 (15.7)
The individual is not moving	45 (15.0)
Others	10 (3.3)

^a^More than one participant gave more than one answer

### Recognizing cardiac arrest

Table 3 shows comparisons between those who had received training about CPR and those who had not regarding cardiac arrest signs, including (a) consciousness evaluation, (b) respiratory evaluation, and (c) circulation evaluation. Based on the table, participants who received training were more likely to know the three signs of consciousness evaluation than those who did not. For respiratory evaluation, the number of participants who received training and did not know that “no steam coming up in front of the mouth of the victim” was an incorrect answer was lower than the number of participants who did not receive training and did not know this answer. For circulation evaluation, the number of participants who had CPR training and could correctly feel a pulse in the vessels of the neck was higher than the number who did not receive training and could feel a pulse.

**Table 3:** Identification of cardiac arrest

Finding	Received training n (%)	Did not receive training n (%)	Total n (%)	*P* value
Consciousness Evaluation				
No response when called^a^	47 (15.7)	84 (28)	131 (43.7)	.015
No response when touched^a^	32 (10.7)	78 (26)	110 (36.7)	NS
No movement^a^	32 (10.7)	64 (21.3)	96 (32.0)	NS
Those who knew the 3 findings correctly	18 (6.0)	16 (5.3)	34 (11.3)	0.04
Those who knew 2 of the findings correctly	21 (7.0)	31 (10.3)	52 (17.3)	.036
Those who knew one of the findings correctly	78 (26.0)	181 (60.3)	259 (86.3)	NS
Respiratory Evaluation				
No respiratory movement^a^	36 (12.0)	94 (31.3)	130 (43.3)	NS
No respiratory sound^a^	31 (10.3)	63 (21.0)	94 (31.3)	NS
No air coming out from the mouth^a^	29 (9.7)	70 (23.3)	99 (33)	NS
No steam coming up in front of the mouth of the victim^b^	13 (4.3)	55 (18.3)	68 (22.7)	.027
Those who knew the 3 findings correctly	11 (3.7)	23 (7.7)	34 (11.3)	NS
Those who knew 2 of the findings correctly	13 (4.3)	43 (14.3)	56 (18.7)	NS
Those who knew one of the findings correctly	72 (24.0)	161 (53.7)	233 (77.7)	NS
Circulation Evaluation				
Lack of circulation signs^a^	25 (8.3)	73 (24.3)	98 (32.7)	NS
Not feeling a pulse in the vessels of the neck^a^	50 (16.7)	92 (30.7)	142 (47.3) (47.3).017	.017
Not feeling a pulse in the vessels of the arm^a^	91 (30.3)	28 (9.3)	119 (39.7)	NS
Those who knew the 3 findings correctly	4 (1.3)	13 (4.3)	17 (5.7)	NS
Those who knew 2 of the findings correctly	13 (4.3)	32 (10.7)	45 (15)	NS
Those who knew one of the findings correctly	86 (28.7)	211 (70.3)	297 (99.0)	NS

^a^True reply, NS: Not significant

^b^False reply

Seventy participants had witnessed a sudden cardiac arrest (23.3%). Among those, 42.8% called the ambulance, 20% told someone to call for help, 10% gave chest compression, 10% gave mouth-to-mouth breathing, 8.6% gave both chest compression and mouth-to-mouth breathing, and 8.6% just watched and left. The number of participants who received training and performed chest compression, mouth-to-mouth ventilation, and both massage and ventilation was higher than the number who performed these actions and did not receive training (Table 4).

**Table 4:** Classification of participants who witnessed sudden cardiac arrest by training

Action	Received training n(%)	Did not receive training n (%)	Total n (%)	*P* value
Conduct only chest compression	5 (7.1)	2 (2.9)	7 (10.0)	.007
Conduct only mouth-to-mouth ventilation	5 (7.1)	2 (2.9)	7 (10.0)	.007
Conduct both, chest compression and ventilation	5 (7.1)	1 (1.4)	6 (8.6)	.01

### CPR knowledge and skills

Overall, 37% of the sample knew how to perform chest compression if they witnessed a sudden cardiac arrest. Moreover, 74.3% indicated that they could give both chest compression and ventilation. Regarding mouth-to-mouth ventilation, 47.7% of the sample said that they would perform it if they witnessed a sudden arrest. The number of participants who received training about CPR and knew the correct answers regarding the meaning of chest compression, heart rate, and the massage/ventilation ratio was higher than the number who knew the correct answers and did not receive training (Table 5).

**Table 5:** Practical application of CPR between those who received training and those who did not

Item	Received training n(%)	Did not receive training n(%)	Total n (%)	*P* value
Chest compression meaning^a^	37 (12.3)	51 (17.0)	88 (29.3)	.001
Massage rate^a^	37 (12.3)	41 (13.7)	78 (26)	.000
Massage location^a^	31 (10.3)	56 (18.7)	87 (29)	NS
Massage depth^a^	29 (9.7)	77 (25.7)	106 (53.3)	NS
Massage/ventilation ratio^a^	33 (11.0)	30 (10.0)	63 (21.0)	.000

*NS* not significant

^a^Numbers reflect correct answers

### CPR attitude

When the participants were asked “To whom would you provide CPR without hesitation?” the responses were as follows: 265 (88.3%) someone from the family, 110 (36.7%) friends, and 90 (30%) neighbors.

### Concerns regarding CPR

The three most important concerns regarding CPR application for family members were (1) making a mistake (*n* = 185, 61.7%), (2) causing bone fractures (*n* = 51, 17%), and (3) stopping the heart (*n* = 49, 16.3%). The three most important concerns regarding CPR applications for strangers were (1) making a mistake (*n* = 177, 59%), (2) punishment because of legal reasons (*n* = 87, 29%), and (3) causing harm to organs (*n* = 72, 24%).

## Discussion

CPR knowledge and skills are of great importance to the public and communities, as they are life-saving skills from which all members of society benefit. There have been no previous studies in Jordan concerning public knowledge and attitudes towards CPR. The participants were chosen from diverse and representative parts of Jordan, from among the lay population and different backgrounds. The participants’ characteristics in this study were comparable to those in other similar and related studies [1]. Regarding CPR training, the results showed that 29% of the participants stated that they had received CPR training, higher than that reported in Hong Kong (21%) [15] and mainland China (25.6%) [19] and comparable to data reported elsewhere, including 27% in New Zealand [14] and 28% in Ireland [13]. Reported percentages from other countries, including Australia [11], Poland [10], and the US [9] were higher, with percentages of 58, 75 and 79%, respectively. In general, Jordan has a lower percentage of trained people in comparison to other countries, indicating that more focus is needed to train the public in life-saving skills to bring Jordan in line with international norms.

The results of this study reflect that the main sources of information and training are schools, followed by universities, and finally, the media (i.e., television and the internet). Studies conducted in nearby countries, such as Saudi Arabia, indicate that the most common sources of information on CPR are television and movies [17]. Although schools and universities are of great importance to increase information, the media can reach a broader population and can also cover a large number of people to raise more awareness. Inclusion of such simple training programs by trained personnel could have positive outcomes for public health and communities.

Participants in this study who received training were knowledgeable regarding the evaluation of the three signs of consciousness. Regarding respiratory evaluation, the number of participants who received training and did not know that “no steam coming up in front of the mouth of the victim means they have stopped breathing” is an incorrect answer was lower than that among those who did not receive training. For circulation evaluation, the number of participants who received CPR training and knew how to correctly feel the pulse in the vessels of the neck was higher than the number with this knowledge who did not receive training. These results highlight the importance of training programs and ensuring that necessary information is both fully understood and practiced by trainees.

The response rates for the signs of cardiac arrest showed that the highest rates were for chest pain, respiratory standstill, loss of consciousness and difficulty breathing. Similar data were reported in a study conducted in Turkey, in which respondents indicated that the highest rates were for signs of cardiac arrest (60.7%), followed by 49.3% who answered cessation of circulation, and finally cessation of breathing (40.7%) [1]. Therefore, chest pain and circulation are given first priority by lay people, who then look for respiration. This is in line with the new guidelines of the AHA (to perform chest compression, then attend to breathing, and finally circulation).

In this study, the participants who witnessed sudden cardiac arrest comprised 23.3% of the total sample. Among those, nearly half stated that they called the ambulance, and the rest with equal or lower percentages responded that they called for help, gave chest compression, and gave mouth-to-mouth breathing. The lowest responses were for chest compression and mouth-to-mouth breathing and ‘just watched and left’. These results reflect the nature of Jordanian culture and how people are willing to help each other, especially during emergency situations. However, it is culturally insensitive to give mouth-to-mouth breathing, especially between men and women.

In this study, the numbers of participants who received training and mentioned that they performed chest compression, mouth-to-mouth ventilation, and both massage and ventilation were greater than those who performed these actions and did not receive training. The majority of the respondents (74.3%) indicated that they can give both chest compression and ventilation. The number of participants who received training about CPR and knew the correct answers regarding the meaning of chest compression, heart rate, and the massage/ventilation ratio was higher than the number who knew the correct answers and did not receive training. Training sessions were beyond the scope of the current study; we merely asked participants if they had attended training or not. However, such information forms the basics of any CPR training course, even if it is learned through the media. These results indicate the importance of training programs, which empower people to provide help in emergency situations.

A high percentage of participants in this study showed no hesitation to provide CPR to their family members; the next highest percentage was to friends and finally, to neighbors. Concerns regarding CPR applications for family and strangers were classified in priority order. For family members, the highest percentage of concern was making mistakes (61.7%), and the lowest was punishment because of legal reasons (15%). Concerns regarding performing CPR on strangers were classified in priority order from making a mistake, to concern of punishment because of legal reasons (59%), with the least concern about causing a bone fracture (18.3%). The lowest priority concern for performing CPR to both family and strangers was contamination by blood or vomit. This indicates that life-saving attempts for family members and strangers will be given high priority, with apprehension about making mistakes being the most important concern. These results indicate the high necessity of education and training programs for the community.

Chair et al. [15] mentioned that people with full-time jobs and higher levels of education were more likely to have received CPR training. Respondents stating they had received CPR training were more willing to try it if needed at home (odds ratio = 3.3; 95% confidence interval, 2.4–4.6; *P* < 0.001), and on strangers in the street (4.3; 3.1–6.1; *P* < 0.001) in case of emergencies. Overall, the CPR knowledge of the respondents was low (median = 1, out of 8). More than 90% of the sample in this study was employed, and more than half of the sample also had diplomas and higher degrees of education. These characteristics support the idea of a greater willingness to conduct CPR among educated and employed people.

In their study, Chen et al. [19] described the concerns regarding CPR applications for family members and strangers. The questionnaire included individual information, current status of bystander CPR training, and individuals’ willingness and attitudes towards performing CPR. The authors found that 25.6% of lay people received CPR training, the majority (98.6%) of whom would perform CPR on their family members, but fewer (76.3%) were willing to perform it on strangers. Most respondents (53.2%) were worried about legal issues. When laws were implemented to protect bystanders who give first aid, the number of laypersons who were not willing to perform CPR on strangers dropped from 23.7 to 2.4%. Although the number of people in China who knew CPR was increasing over time, CPR training was still much less common than in many developed countries. The barriers as mentioned by the authors were that laypersons were not well trained, and they feared being prosecuted for unsuccessful CPR. The authors also recommended a need for more accredited CPR training courses, and they noted that laws should be passed to protect bystanders who provide assistance.

There are some centers providing CPR training for lay persons in different areas in Jordan, including in the north, middle, and south. However, to date, there is no legislation on the part of the Ministry of Health and the Ministry of Interior to protect laypersons in case they perform CPR on victims and mistakes occur. For this reason, some people will hesitate to provide CPR if the victim is not one of their family members. The relevant government bodies should be informed about such results in order to improve outcomes and decrease mortality and morbidity after cardiac arrest, especially OHCA.

## Conclusion and recommendations

The results of this study indicate the importance of continuing to train the population in CPR knowledge and skills; specifically, CPR education programs and evaluations of their effectiveness are needed. The media can play a role in increasing the knowledge of the public, hence the authors recommend promoting the training programs in the media, utilizing cheap and effective technologies such as social media to reach the general population, as well as providing more formal instruction through schools and universities. In addition, national bylaws should protect those who provide assistance to people with heart failure in such cases.

### Limitations

The major limitation of this study was the use of a cross-sectional design with a convenience sampling method. Moreover, we used the 2010 AHA criteria and did not ask about the timing for when participants received their training.

## Additional file

Additional file 1: Questionnaire. Description of data: Data collection instrument in Arabic and English. (DOCX 30 kb)

## Abbreviations

AED: Automated external defibrillator
AHA: American Heart Association
BLS: Basic life support
CPR: Cardiopulmonary resuscitation
OHCA: Out-of-hospital cardiac arrest
SPSS: Statistical package for social sciences

## References

[1] Ozbilgin S, Akan M, Hanci V, Aygun C, Kuvaki B. Evaluation of public awareness, knowledge and attitudes about cardiopulmonary resuscitation: report of Izmir. Turk J Anaesthesiol Reanim 2015;43(6):396–405. Epub 2016/07/02. doi: 10.5152/TJAR.2015.61587. PubMed PMID: 27366536; PubMed Central PMCID: PMC4894183. 10.5152/TJAR.2015.61587 PMC4894183 27366536

[2] Puttgen HA, Pantle H, Geocadin RG. Management of cardiac arrest patients to maximize neurologic outcome. Curr Opin Crit Care 2009;15(2):118–124. Epub 2009/07/07. doi: 10.1097/MCC.0b013e328326077c00075198-200904000-00008. PubMed PMID: 19578322. 10.1097/MCC.0b013e328326077c 19578322

[3] Berdowski Jocelyn, Berg Robert A., Tijssen Jan G.P., Koster Rudolph W. (2010). Global incidences of out-of-hospital cardiac arrest and survival rates: Systematic review of 67 prospective studies. Resuscitation.

[4] Benjamin EJ, Blaha MJ, Chiuve SE, Cushman M, Das SR, Deo R, et al. Heart Disease and Stroke Statistics-2017 Update: A Report From the American Heart Association. Circulation. 2017;135(10):e146-e603. Epub 2017/01/27. doi: 10.1161/CIR.0000000000000485. PubMed PMID: 28122885; PubMed Central PMCID: PMC5408160. 10.1161/CIR.0000000000000485 PMC5408160 28122885

[5] Berdowski Jocelyn, Blom Marieke T., Bardai Abdennasser, Tan Hanno L., Tijssen Jan G.P., Koster Rudolph W. (2011). Impact of Onsite or Dispatched Automated External Defibrillator Use on Survival After Out-of-Hospital Cardiac Arrest. Circulation.

[6] McNally B, Robb R, Mehta M, Vellano K, Valderrama AL, Yoon PW, et al. Out-of-hospital cardiac arrest surveillance --- cardiac arrest registry to enhance survival (CARES), United States, October 1, 2005--December 31, 2010. MMWR Surveill Summ 2011;60(8):1–19. Epub 2011/07/29. PubMed PMID: 21796098. 21796098

[7] Rajapakse Renata, Noč Marko, Kersnik Janko (2010). Public knowledge of cardiopulmonary resuscitation in Republic of Slovenia. Wiener klinische Wochenschrift.

[8] Mpotos Nicolas, Vekeman Eva, Monsieurs Koenraad, Derese Anselm, Valcke Martin (2013). Knowledge and willingness to teach cardiopulmonary resuscitation: A survey amongst 4273 teachers. Resuscitation.

[9] Sipsma Kristen, Stubbs Benjamin A., Plorde Michele (2011). Training rates and willingness to perform CPR in King County, Washington: A community survey. Resuscitation.

[10] RASMUS A., CZEKAJLO M. S. (2000). A national survey of the Polish populationʼs cardiopulmonary resuscitation knowledge. European Journal of Emergency Medicine.

[11] Celenza Tony, Gennat Hanni C, O'Brien Debra, Jacobs Ian G, Lynch Dania M, Jelinek George A (2002). Community competence in cardiopulmonary resuscitation. Resuscitation.

[12] Kuramoto Nobuo, Morimoto Takeshi, Kubota Yoshie, Maeda Yuko, Seki Susumu, Takada Kaori, Hiraide Atsushi (2008). Public perception of and willingness to perform bystander CPR in Japan. Resuscitation.

[13] Jennings Siobhan, Hara Tom O., Cavanagh Brendan, Bennett Kathleen (2009). A national survey of prevalence of cardiopulmonary resuscitation training and knowledge of the emergency number in Ireland. Resuscitation.

[14] Larsen P, Pearson J, Galletly D. Knowledge and attitudes towards cardiopulmonary resuscitation in the community. N Z Med J 2004;117(1193):U870. Epub 2004/05/11. PubMed PMID: 15133520. 15133520

[15] Chair SY, Hung MS, Lui JC, Lee DT, Shiu IY, Choi KC. Public knowledge and attitudes towards cardiopulmonary resuscitation in Hong Kong: telephone survey. Hong Kong Med J. 2014;20(2):126–133. Epub 2014/03/15. doi: 10.12809/hkmj134076. PubMed PMID: 24625387. 10.12809/hkmj134076 24625387

[16] Coons Stephen Joel, Guy Mignonne C. (2009). Performing bystander CPR for sudden cardiac arrest: Behavioral intentions among the general adult population in Arizona. Resuscitation.

[17] Al-Turki YA, Al-Fraih YS, Jalaly JB, Al-Maghlouth IA, Al-Rashoudi FH, Al-Otaibi AF, et al. Knowledge and attitudes towards cardiopulmonary resuscitation among university students in Riyadh, Saudi Arabia. Saudi Med J. 2008;29(9):1306–1309. Epub 2008/09/25. PubMed PMID: 18813417. 18813417

[18] Al-Najjar T, Al-Khouli AT, Dakak FM, Awad JA, Naseef MA, Kentab OY (2011). Arabic Version of Basic Life Support for Health Care Providers.

[19] Chen Meng, Wang Yue, Li Xuan, Hou Lina, Wang Yufeng, Liu Jie, Han Fei (2017). Public Knowledge and Attitudes towards Bystander Cardiopulmonary Resuscitation in China. BioMed Research International.

