# Supplementary material for: Evaluation of public awareness, knowledge and attitudes towards basic life support: a cross-sectional study
Source: BMC Emerg Med. 2018 Oct 29;18:37. doi: 10.1186/s12873-018-0190-5 (PMC6206630; doi:10.1186/s12873-018-0190-5)
Supplement: Supplementary file 1 — Questionnaire. Description of data: Data collection instrument in Arabic and English. (DOCX 30 kb) [file 12873_2018_190_MOESM1_ESM.docx]

Additional file 1

استبانة "تقييم الوعي المجتمعي والمعرفة والإتجاهات نحو أساسيات إنقاذ الحياة"

تتكون الإستبانة من جزأين:

أ.المعلومات الشخصية.

ب.أسئلة عن معرفتك ووجهة نظرك حول استخدام طرق إنقاذ الحياة.

أ.المعلومات الشخصية:

- الرجاء كتابة العمر:

*الرجاء وضع إشارة (X) في المربع المناسب عند الأسئلة التالية:

-المشارك : □ ذكر □ أنثى

-الحالة الإجتماعية : □متزوج □ أعزب □ مطلق

- مستوى التعليم : □ إبتدائي □ إعدادي □ ثانوي

□ دبلوم □ بكالوريوس □ دراسات عليا

-الوظيفة : □ وظيفة حكومية □ وظيفة قطاع خاص □ لدي عمل خاص بي

□ متقاعد □ ربة منزل □ طالب □ مزارع

□ لا يعمل

ب.أسئلة عن معرفتك ووجهة نظرك حول معلومات تتعلق بأساسيات إنقاذ الحياة :

*الرجاء وضع إشارة (X) في المربع المناسبعند الأسئلة التالية ، ويمكنك إختيار أكثر من إجابة للسؤال الواحد.

1- أي من التالية تعتبر من علامات السكتة القلبية ؟

□ فقدان الوعي

□ توقف النفس

□ توقف الدورة الدموية (لا يسمع دقات القلب ولا يشعر بالنبض)

□ إزرقاق

□ الشعور باللعيان (غثيان)

□ وجع بالصدر

□ ضعف في الجسم

□ الشخص لا يتحرك

□ أخرى

2- كيف يمكن تحديد درجة وعي أو إفاقة الشخص :

□ لا استجابه عند المناداه

□ لا استجابه عند لمسه

□ لا يتحرك اطلاقاً

□ لا أعرف

3- كيف يمكن تحديد توقف التنفس؟

□ لا يوجد أي حركة بالنفس

□ لا يوجد أي صوت للتنفس

□ لا يخرج هواء من فم الشخص

□ لا يظهر بخار عند وضع المرآه أمام الهواء الخارج من فم الشخص

□ لا أعرف

4- كيف يمكنك تحديد توقف الدورة الدموية؟

□ علامات قلة الدورة الدموية

□ لا يمكنك جس النبض عند أوعية الرقبة

□ لا يمكنك جس النبض عند أوعية اليد

5- هل شهدت وفاة مفاجئة ؟ إذا نعم من هو /هي من التالية ؟

□ أحد أفراد عائلتي

□ أحد الأصدقاء أو المعارف

□ شخص غريب

□ لم أرى تلك الحالة

6- إذا شهدت مثل الحالة السابقة (الوفاة المفاجئة) ، ماذا فعلت بتلك الحالة؟ (إذا لم تشهد هذهالحالةلا تجب عن هذا السؤال )؟

□ بدأت بعمل تدليك للقلب

□ قمت بعمل إنعاش فموي (من الفم للفم)

□ قمت بعمل تدليك للقلب إضافة إلى الإنعاش الفموي (انعاش قلبي رئوي)

□ اتصلت بالدفاع المدني (911)

□ طلبت من الآخرين طلب المساعدة

□ طلبت المساعدة عبر الهاتف / الموبايل

□ فقط شاهدت الموقف وغادرت

7- ماذا تعتقد المعنى الصحيح لعبارة (تدليك القلب)؟

□ تدليك القلب على فترات معينة

□ الضغط القوي على الصدرعلى فترات معينة

□ تدليك القلب مباشرة بعد كشف منطقة الصدر

□ الضغط على القلب مباشرة بعد كشف منطقة الصدر

□ ليس لدي أي فكرة عن المعنى

8- في حال حدثت وفاة مفاجئة أمامك ، أي من الأشخاص التالية سوف تقدم لهم التنفس وتدليك القلب ؟

□ أحد أفراد عائلتك

□ أصدقائك

□ جيرانك

□ الشاب/الشابة في الصالة الرياضية

□ أي شخص غريب في السوبرماركت أو مكان التسوق

□ الشخص المتسخ والذي لا يبدو نظيفاً في موقف الباص

□ شخص مدمن على المخدراتوالحشيش والأدوية

9- إذا فقد أحد الأفراد من عائلتك أو أصدقائك الوعي (موت مفاجىء) ، ماذا تفعل ؟

□ أبدأ بعمل تدليك القلب

□ أتصل بالدفاع المدني (911)

□ أتصل بأشخاص أعرفهم أو أتصل لطلب المساعدة

□ أراقب فقط ثم أغادر

10- ماذا تفعل إذا شاهدت شخص غريب عنك فاقد الوعي (الموت الفجائي)؟

□ أبدأ بعمل تدليك القلب

□ أتصل بالدفاع المدني (911)

□ أتصل بأشخاص أعرفهم أو أتصل أطلب المساعدة

□ أراقب فقط ثم أغادر

11- ما هي الإعتبارات /المخاوف التي تمنعك من تقديم تدليك القلب لأصدقائك أو أقاربك ؟

□ إرتكاب خطأ

□ التسبب بكسر بالعظم

□ التسبب بأذى لأعضاء الجسم

□ إيقاف عمل القلب

□ العقاب بسبب التشريعات /القوانين السائدة

□ التلوث من الدم والقيء

□ التعرض لمرض معدي

□ أخرى

12- ما هي الإعتبارات / المخاوف التي تمنعك من تقديم تدليك القلب للشخص الغريب؟

□ إرتكاب خطأ

□ التسبب بكسر بالعظم

□ التسبب بأذى لأعضاء الجسم

□ إيقاف عمل القلب

□ العقاب بسبب التشريعات /القوانين السائدة

□ التلوث من الدم والقيء

□ التعرض لمرض معدي

□أخرى

13- هل تعرف كيف تقدم تدليك القلب في حالة توقف القلب وتوقف التنفس (الموت المفاجىء)؟

□ نعم

□ لا

14- هل تلقيت أي تدريب بهذا الموضوع (أساسيات إنقاذ الحياة)؟

□ نعم

□ لا

15- إذا كانت إجابتك نعم عن السؤال السابق ، أين تلقيت التدريب؟

□ في المدرسة

□ في الجامعة

□ في الخدمة العسكرية

□ أماكن تعليم السواقة

□ مؤسسات تعليم الإنعاش القلبي الرئوي

□ مدرب من وزارة الصحة

□ دورة تدريبية من البلدية

□ في النادي الرياضي

□ في مكان العمل

□ وسائل الإعلام ، التلفاز ، الإنترنت

□ أخرى

16- إذا واجهت شخص توقف قلبه عن العمل ، أي من طرق إنقاذ الحياة التالية سوف تستخدم ؟

□ فتح المجرى التنفسي

□ السيطرة على التنفس

□ أقدم الإنعاش الفموي (من الفم للفم)

□ أقوم بتدليك القلب

□ أقدم الإنعاش الفموي وتدليك القلب معاً

□ لا أعرف

17-ما هو الرقم الصحيح لمعدل تدليك القلب مقابل التنفس (تدليك القلب / التنفس)؟

□ 5/1

□ 15/2

□ 30/2

□ أخرى

18- أي منطقة من الصدر يمكنك عمل تدليك للقلب فيها ؟

□ الجزء العلوي من الصدر

□ الجزء الأوسط من الصدر

□ الجزء الأسفل من الصدر

□ أخرى

19- ما هو معدل تدليك القلب ؟

□ 150 مرة/الدقيقة على الأقل

□ 100 مرة/الدقيقة على الأقل

□ 50مرة /الدقيقة على الأقل

□ لا أعرف

20- ما هي شدة الضغطة على القلب ؟

□ ضغطة بسيطة ، بما يكفي لجعل القفص الصدري يتحرك إلى الأسفل 1-2 سم

□ ضغطة معتدلة ، لجعل القفص الصدري يتحرك إلى الأسفل 5-6 سم

□ ضغطة شديدة ، لجعل القفص الصدري يتحرك إلى الأسفل 6-10 سم

□ أكبر قدر ممكن من قوة الضغط

□ أخرى

21- ماذا تعرف عن جهاز "الصدمات الكهربائية" الذي يستخدم لتدليك القلب عند الحاجة؟

□ لم أسمع به من قبل

□ سمعت عنه من قبل ولم أره

□ هو جهاز لدعم التنفس

□ هو جهاز لتشغيل القلب بعد توقفه

22- هل لديك فكرة أين يتواجد جهاز "مزيل الرجفان الخارجي الآلي " أو "جهاز تنظيم ضربات القلب "؟

□ نعم

□ لا أعلم

**A- Demographic information:**

**Please state your age:**

**Please state your gender:**  Male  Female

**Please state your marital status:**  I am married  I am not married  I am divorced

**Please state your educational status:**  Primary school  College

 Secondary School  University

 High School  Post graduate

**Please state your occupation:**

 Civil Servant  Retired

 Worker  House wife

 Self-employed  Student

 Unemployed  Farmer

**1) Which of the following may be a sign of sudden cardiac arrest? (You can mark more than one option)**

 Loss of consciousness (the individual appearing to have fainted)

 Discontinuation of breathing (respiratory standstill)

 Discontinuation of circulation (the heart sound not being heard or not feeling the pulse)

 Cyanosis

 Nausea

 Chest pain

 Faintness of the skin

 The individual is not moving

 Other

**2) How can the consciousness state of the individual be determined? (You can choose more than one option)**

 No response when called

 No response when touched

 Not moving at all

 I do not know

**3) How can the absence of respiration be determined? (You can choose more than one option)**

 Not having any respiratory movement

 Not having any respiratory sound

 Not coming air out of the mouth of individual

 Not steaming up a mirror placed in front of the mouth of individual

 I do not know

**4) How can the absence of circulation be determined? (You can choose more than one option)**

 The lack of circulation signs

 Not feeling a pulse in the vessels of the neck

 Not feeling a pulse in the vessels of the arm

 I do not know

**5) Have you ever witnessed a sudden death? If yes, who was he/she? (You can choose more than one option)**

 Somebody from my family

 Somebody from my friends or acquaintances

 A stranger

 I have not seen this

**6) If you have witnessed such an event, what did you do in the situation? (If you replied no to the 4th question, skip this question)**

**(You can choose more than one option)**

 I began to give cardiac massage

 I conducted mouth to mouth ventilation (I respirated)

 I both gave cardiac massage and conducted mouth to mouth ventilation (I gave CPR)

 I called an ambulance (911)

 I told somebody to call for help

 I called for help by telephone

 I just watched and left

**7) What do you think a “cardiac massage” means?**

 To scrub the chest at certain intervals

 To apply strong compression to the chest at certain intervals (compress)

 To scrub the heart directly opening the chest wall

 To apply compression directly to the heart opening the chest wall

 I have no idea

**8) If sudden death occurs in the following people, for whom would you conduct respiration and give cardiac massage?**

**(You can choose more than one option)**

 Someone from the family

 Your friend

 Your neighbour

 A youth in the sports hall

 A stranger in the supermarket

 A person who has poor personal hygiene at the bus stop

 A gamin who is drug dependent and uses glue, hashish and heroin

**9) If somebody from among your family members or friends felt faint (sudden death) what would you do?**

 I would begin to give cardiac massage

 I would call an ambulance (911)

 I would call somebody or call for help

 I would just watch and leave

**10) What would you do if you witness a stranger feeling faint (sudden death)?**

 I would begin to give cardiac massage

 I would call an ambulance (911)

 I would call somebody or call for help

 I would just watch and leave

**11) What concerns may prevent you from giving cardiac massage to your friends or relatives?**

 Making a mistake

 Causing bone fractures

 Causing harm to organs

 Stopping a working heart

 Punishment due to legal reasons

 Contamination by blood or vomit

 Contracting a contagious disease

 Other

**12) What concerns may prevent you from giving cardiac massage to a stranger?**

 Making a mistake

 Causing bone fractures

 Causing harm to organs

 Stopping a working heart

 Punishment due to legal reasons

 Contamination by blood or vomit

 Contracting a contagious disease

 Other

**13) Do you know how to give cardiac massage in the case of cardiac arrest and respiratory standstill (namely, sudden death)?**

 Yes

 No

**14) Have you received any training in this subject? (Basic Life Support training)**

 Yes

 No

**15) If your reply is yes to the above question, where did you receive the training?**

 At school

 At university

 During my military service

 During the driving school

 At a Resuscitation Society course

 At a course given by the trainers of the Ministry of Health

 At a course given by the municipality

 In a sports club

 At a course given in the workplace

 Television-Internet-Media

 Other

**16) If you are confronted with a person whose heart has stopped, which of the Basic Life Support applications can you apply?**

**(You can choose more than one option)**

 I can open the airway

 I can control respiration

 I can ventilate/conduct mouth to mouth ventilation (kiss of life)

 I can give cardiac massage

 I can both ventilate and give cardiac massage

 I do not know

**17) What is the proper rate of cardiac massage/artificial ventilation during cardiac massage?**

 5/1

 15/2

 30/2

 Other

**18) Which area must cardiac massage be applied on?**

 Upper part of the chest

 Middle of the chest

 Lower part of the chest

 Other

**19) What must be the rate of the cardiac massage?**

 At least 150 times per minute

 At least 100 times per minute

 At least 50 times per minute

 I do not know

**20) How much force must be applied during heart massage?**

 Enough that the rib cage moves down 1 to 2 cm

 Moderate force, such that the rib cage moves down 5 to 6 cm

 High force, such that the rib cage moves down 6 to 10 cm

 As much force as possible

 Other

**21) What do you know about the device defined as a “defibrillator” that is used during cardiac massage when necessary?**

**(You can choose more than one option)**

 I have never heard of it

 I have heard of it before but have not seen it

 It is a device supporting respiration

 It is a device to restart a heart that has stopped working

**22) Do you have any idea about where an “Automated External Defibrillator” or “Pace Maker” can be found?**

 Yes

 I do not know
